# Prevention of mother-to-child transmission of HIV service interruptions amid COVID-19 pandemic

**DOI:** 10.4102/safp.v66i1.5899

**Published:** 2024-05-13

**Authors:** Florence M.Q. Setshedi, Livhuwani Tshivhase, Idah Moyo

**Affiliations:** 1 Department of Nursing, Faculty of Healthcare Sciences, Sefako Makgatho Health Sciences University, Pretoria, South Africa; 2 Department of Nursing, Faculty of Health Sciences, Sefako Makgatho Health Sciences University, Pretoria, South Africa; 3 Department of Health Studies, College of Human Sciences, University of South Africa, Pretoria, South Africa; 4 Department of HIV Services, Populations Solutions for Health, Harare, Zimbabwe

**Keywords:** COVID-19, descriptive phenomenology, HIV, interruptions, PMTCT services

## Abstract

**Background:**

The coronavirus disease 2019 (COVID-19) caused global disruptions in healthcare service delivery. The prevention of mother-to-child transmission (PMTCT) of human immunodeficiency viruses (HIV) services were also interrupted, threatening the attainment of Sustainable Development Goal 3. This article describes the PMTCT service interruptions experienced during the COVID-19 pandemic in Tshwane healthcare facilities.

**Methods:**

A descriptive phenomenological design was used to explore and describe the experiences of healthcare providers offering PMTCT services during COVID-19 in the Tshwane district, Gauteng province. Purposive sampling was used to recruit participants. Data were collected through in-depth interviews with 16 participants, and Colaizzi’s data analysis steps were followed in analysing the findings.

**Results:**

Participants reported interruptions in PMTCT service delivery during the pandemic. Non-adherence to scheduled visits resulted in patients defaulting or not adhering to treatment regimens, high viral loads and mother–infant pairs’ loss to follow-up. Other features of service disruption included late antenatal bookings, low client flow and delays in conducting deoxyribonucleic acid-polymerase chain reaction (DNA-PCR) testing in HIV-exposed babies. In addition, staff shortages occurred because of re-assignments to COVID-19-related activities. Study participants were psychologically affected by the fear of contracting COVID-19 and worked in a frustrating and stressful environment.

**Conclusion:**

Improved community-based follow-up services are critical to enhance PMTCT service outcomes and prevent infant HIV infections.

**Contribution:**

The findings may influence policymakers in developing strategies to curb HIV infections among mothers and children during pandemics.

## Introduction

To promote the prevention of mother-to-child transmission (PMTCT) of human immunodeficiency viruses (HIV), HIV-positive mothers receive a service from healthcare providers during pregnancy, labour, delivery or breastfeeding to prevent their child from contracting HIV.^1^ The PMTCT services entail women accessing antenatal care (ANC), HIV counselling and testing (HCT), education on safe delivery and infant feeding, follow-up 3 days post-delivery, HIV testing at the 7-day postnatal visit, family planning services and lifelong antiretroviral treatment (ART).^2^

Approximately 5% – 10% of HIV infections are transmitted during pregnancy, 10% – 15% during childbirth, and 5% – 20% during the breastfeeding period.^3^ Prevention of mother-to-child transmission services were thus initiated to curb HIV transmission to newborns. According to Rahmadhani,^4^ PMTCT is considered a successful strategy to reduce the risk of mothers transmitting HIV to their infants if it is implemented properly and thoroughly. The World Health Organization (WHO)^1^ reports that the transmission of HIV from the mother to the child could be prevented by taking ART during pregnancy and lactation. However, the prevalence of women aged 15–49 years who are infected with HIV is reported to be increasing, and the number of children with HIV and AIDS is consequently rising too.^5^ Thus, there is a need to reduce the rate of mother-to-child transmissions in South Africa and globally. To ensure the PMTCT programme’s effectiveness, mothers and babies require antenatal services and HIV testing during pregnancy, access to ART, safe childbirth practices, infant feeding, infant HIV testing and other postnatal healthcare services.^6^ Challenges commonly seen as affecting PMTCT strategy implementation are a lack of information about ART, insufficient family support, the severity of healthcare workers’ workload, limited HIV test equipment and drug stockouts.^4^

According to guidelines, babies undergo HIV testing at birth, 10 weeks, 6 months, 18 months and 6 weeks after the mother stops breastfeeding. After birth, all exposed babies are placed on preventive treatment and an antibiotic to prevent infections until the mother stops breastfeeding.^7^ However, the emergence of the coronavirus disease 2019 (COVID-19) and the lockdown measures meant to contain the pandemic created various challenges for healthcare workers and recipients of PMTCT care.^8^

According to Murewanhema et al.,^9^ access to and the use of ANC services decreased during the COVID-19 pandemic. In addition, there have been reports of home deliveries because of COVID-19 restrictions, service barriers to ANC access and use as a result of movement restrictions, limited transport access, fear of contracting COVID-19 at health facilities and various facility-related barriers.^9^ An Indonesian study established that response activities meant to curb the pandemic were prioritised at the expense of other public health interventions, such as the provision of HIV services.^10^ Moreover, healthcare settings were overwhelmed with a high number of patients and lacked the infrastructure and resources to promote social distancing. Findings from a South African study also outlined that migrants on PMTCT programmes faced treatment interruptions as borders were closed and required official documents for them to receive care.^11^ Bisnauth et al.^11^ thus highlighted the need for multi-month dispensing of long-term ART supplies to facilitate treatment continuity.

During the COVID-19 pandemic, several countries reported significant disruptions in HIV care, impacting the prevention, testing and support given to people living with HIV.^12^ A study conducted in sub-Saharan Africa through social media indicated that 13% of patients on chronic medication could not access their treatment because of COVID-19 restrictions.^13^ In Zimbabwe, 19% of people with HIV were unable to refill their antiretrovirals, while 14% of afflicted individuals in Kenya and Nigeria never collected the needed medication.^6^ The same report indicated that 13 countries in sub-Saharan Africa had 3-month stockouts of first-line ART as suppliers were not supplying hospitals. However, failure to access HIV treatment could ultimately threaten pregnant women’s ability to prevent infecting their unborn child.

Evidence has demonstrated that COVID-19 interrupted paediatric HIV prevention and care.^14^ The same study reported a decrease in pregnant women accessing PMTCT services, increased new paediatric infections and disruptions in caring for children living with HIV. United Nations International Children’s Emergency Fund (UNICEF)^15^ also concurred on the limited implementation of PMTCT guidelines because of the impact of COVID-19. The effectiveness of the PMTCT programme is critical in the reduction of under-five morbidity and mortality. In addition, the PMTCT programme plays a pivotal role in HIV epidemic control and in the attainment of Sustainable Developmental Goal 3 which focuses on health and wellbeing. Against this background, this study explored the experiences of professional nurses and mentor mothers who provided PMTCT care during the COVID-19 pandemic.

## Research method and design

A descriptive phenomenological study was conducted to explore the experiences of professional nurses and mentor mothers involved with the PMTCT of HIV during the COVID-19 pandemic. This design was chosen for its ability to delve deep into participants’ experiences as they had lived it.^16^ The design promotes an understanding of participants’ subjective experiences and offers insights into people’s actions and motivations.^17^ The primary researcher’s bias and preconceptions were kept neutral so the study’s objectives would not be influenced. The researcher thus bracketed some of her opinions, beliefs and experiences during the interviews to add rigour to the study.

### Setting

The study was conducted in Tshwane primary health care facilities. The city of Tshwane is the single-largest metropolitan municipality in the country, comprising seven regions and 105 wards. The study was conducted in one region of Tshwane North in the northern district, which has 21 primary health care facilities. This study was conducted in three primary health care facilities that offer the following services: antenatal and postnatal care, HCT, PMTCT, curative and chronic care, emergency care, tuberculosis (TB) treatment, immunisation, integrated child management services, family planning and emergency services. The choice of the three facilities was based on the high number of patients accessing these facilities compared to the rest of the facilities in the city of Tshwane.^18^

### Population and sample

The study population were nurses and mentor mothers working in the selected PMTCT facilities. Purposive sampling was used to select participants for this study, these included six mentor mothers and 10 professional nurses.

The study included professional nurses and mentor mothers who had been providing PMTCT care services in Tshwane healthcare facilities for at least 6 months before the emergence of COVID-19 in March 2020. The study also included all those who met the above-stated criteria, were willing to participate and voluntarily consented to the study. However, all professional nurses and mentor mothers who met the inclusion criteria but were either on leave and/or not willing to participate were excluded from the study. A total of 16 participants formed the sample of the study.

### Data collection

Data were collected after the researcher received relevant from all parties.

Before the interviews, each participant had to voluntarily complete a consent form. The consent was both written and verbal and covered the agreement to be audio recorded during interviews and to publish the findings. Participants’ autonomy assured their voluntary participation at a preferred time and space.

Participants were reassured that the information they shared was confidential and only the researcher and supervisor had access to the interviews. The recordings and transcripts were stored with a password on a computer accessed only by those authorised to do so.

In-depth, individual face-to-face interviews were conducted with 16 purposefully selected participants from 01 March 2023 to 30 April 2023. Data were collected from three purposefully selected clinics based on the high patient headcount in these facilities. The researcher recruited participants and secured a private room for the interviews with the operational manager at the healthcare facilities. Participants’ demographic data were also collected and participants were assigned pseudonyms to ensure anonymity. The interviews were scheduled for lunchtimes, after hours and on participants’ days off when they could be available for interviews, so patient care was not interrupted. The interviews lasted 40 min – 60 min and were guided by the following central question: ‘*What were your experiences providing PMTCT services during the COVID-19 pandemic*?’ Probes were added following the participants’ responses. The researcher had a notebook wherein field notes were written, and interviews were audio-recorded with participants’ permission. The number of participants was determined by saturation, as no new information emerged from the last two interviews.

### Data analysis

Colaizzi’s data analysis steps were used to establish superordinate themes, themes and subthemes.^19^ Audio recordings were transcribed verbatim by the researcher as she collected the data. In the first step, the researcher read all the transcripts repeatedly to become familiar with the data. In the second step, the researcher identified all statements directly relevant to the phenomenon under investigation. For the third step, the researcher identified meanings relevant to the phenomenon by carefully considering significant statements. By bracketing her pre-suppositions, the researcher was able to stick to the phenomenon experienced by participants.

Meanings were clustered into themes in step four, and in step five, the researcher wrote a full and inclusive description of the phenomenon; the researcher developed an exhaustive description of those aspects deemed essential to the structure of the phenomenon. After analysing and developing the themes, the researcher phoned eight participants to verify if the findings reflected their experiences. Themes were then adopted after telephonic confirmation from the participants. The collected data were shared with the third author, an experienced qualitative researcher who independently coded the data. After coding, the two researchers discussed and agreed on the themes that are presented in Table 2.

### Trustworthiness

The researcher ensured the study’s trustworthiness by adhering to the four measures described by Lincoln and Guba,^20^ namely credibility, dependability, transferability and confirmability. Credibility was ensured through member checking as the researcher was able to contact participants to verify if the finding represented their experiences. The researcher observed reflexivity to ensure credibility through self-awareness and guarded against personal bias when conducting interviews. Transferability was attained through a thick description of the research process so that any other researcher could transfer the findings to their own setting. A pilot study was also conducted with three participants who were not part of the main study to ascertain whether the semi-structured interview guide was clear or required alterations. Dependability was ensured by the detailed descriptions of the steps taken in the research study so that another researcher could replicate the process.^21^ Confirmability was enhanced by the use of an independent coder for data analysis, keeping an audit trail of the audio recordings and field notes taken during the interviews.

### Ethical considerations

Ethical clearance to conduct the study was provided by from the Sefako Makgatho Health Sciences University Ethics Committee (reference no.: SMUREC/H/281/2022:PG) on 08 September 2022, permission from the Gauteng Provincial Department of Health, the Tshwane district health directorate and the operational managers of the facilities selected for the study. The study was conducted in accordance with the Helsinki Declaration, as revised in 2013. Participants received information leaflets that explained the purpose and the processes of the study. Participants were also informed that they were allowed to withdraw from the study at any time without penalty. To ensure privacy, interviews were conducted in a private room secured at the facilities. Confidentiality was also promoted by using participants’ pseudonyms when interviewing and recording.

## Results

Participants were professional nurses and mentor mothers involved in providing PMTCT services during the COVID-19 pandemic. Table 1 presents the participants’ demographic data.

**TABLE 1:** Participants’ demographic data.

Number	Pseudonyms	Age (years)	Gender	Category	PMTCT training	Years of work experience
01	Lebo	53	F	Mentor mother	Yes	12 years
02	Mavis	35	F	Professional nurse	Yes	7 years
03	Bokang	25	F	Professional nurse	Yes	3 years
04	Luthando	50	F	Professional nurse	Yes	17 years
05	Promise	42	F	Mentor mother	No	8 Years
06	Agnes	56	F	Professional nurse	Yes	17 Years
07	Thandi	51	F	Professional nurse	Yes	12 years
08	Palesa	48	F	Professional nurse	No	4 years
09	Johanna	52	F	Professional nurse	Yes	10 Years
10	Elisa	54	F	Professional nurse	Yes	22 years
11	Alina	38	F	Professional nurse	Yes	15 years
12	Abigail	38	F	Mentor mother	Yes	5 years
13	Sharon	25	F	Mentor mother	Yes	9 years
14	Mammy	51	F	Mentor mother	Yes	2 years
15	Amukelani	30	F	Mentor mother	Yes	5 years
16	Khuthadzo	29	F	Professional nurse	Yes	4 years

*Source*: Adapted from Setshedi MFQ. Experiences of healthcare workers rendering prevention of mother-to-child transmission services during the COVID-19 lockdown in Tshwane District Gauteng Province, South Africa. [unpublished dissertation]. Pretoria: Sefako Makgatho Health Sciences University

F, female; PMTCT, prevention of mother-to-child transmission.

This section presents the participants’ experiences while providing care to PMTCT clients during the COVID-19 pandemic. Data analysis yielded the following superordinate themes: non-adherence to scheduled clinic visits, effects of COVID-19 on PMTCT service delivery and clinical barriers. Each superordinate theme contains several themes and subthemes, as presented in Table 2.

**TABLE 2:** Superordinate themes, themes and subthemes.

Superordinate themes	Themes	Subthemes
Non-adherence to scheduled clinic visits	1. Factors associated with non-adherence to clinical appointments	1.1 Missed follow-up PMTCT scheduled clinic visits 1.2 High viral load because of treatment default
	2. PMTCT outcomes	2.1 Loss to follow-up of mother–infant pairs 2.2 Defaulting and non-adherence to ART
Effects of COVID-19 on PMTCT service delivery	3. PMTCT service disruptions	3.1 Late ANC bookings 3.2 Low client flow at PMTCT settings
	4. Interruptions to infant HIV testing	4.1 Delays in conducting DNA-PCR testing in HIV-exposed babies 4.2 HIV-exposed babies’ loss to follow-up
Clinical barriers	5. Resource-related barriers	5.1 Shortage of staff 5.2 Re-assignment of staff
	6. Psychological barriers	6.1 Fear of contracting COVID-19 6.2 Frustrating and stressful work environment

PMTCT, prevention of mother-to-child transmission; COVID-19, coronavirus disease 2019; ART, antiretroviral treatment; ANC, accessing antenatal care; DNA-PCR, deoxyribonucleic acid-polymerase chain reaction.

### Non-adherence to scheduled clinic visits

This superordinate theme focused on the impact of individuals’ non-adherence to scheduled clinic visits. The two themes that emerged included factors associated with non-adherence to clinical appointments and the resultant PMTCT outcomes.

#### Theme 1: Factors associated with non-adherence to clinical appointments

The participants reported that various issues emerged because of non-adherence to clinic appointments. These included missed follow-up PMTCT scheduled clinic visits and high viral loads because of treatment default.

**Subtheme 1.1: Missed follow-up prevention of mother-to-child transmission scheduled clinic visits:** Participants indicated the COVID-19 pandemic resulted in PMTCT clients missing scheduled appointments, as shown in the following extracts:

‘A lot of clients were not complying to their scheduled appointments, people preferred to stay at home rather, they would present to the facility with complication or when in labour.’ (Luthando, 50 years old, female, professional nurse)

‘Some clients presented at the facility after missing several clinic visits, I remember a mother who continued to breast feed the baby who had tested HIV positive twice, only to present with the baby after 2 years and the baby was now HIV positive.’ (Mavis, 35 years old, female, professional nurse)

**Subtheme 1.2: High viral load because of treatment default:** This theme highlights the negative effects that are caused by PMTCT clients defaulting on their ART, as the following excerpts demonstrate:

‘Some clients defaulted taking antiretroviral therapy and never came back for their resupplies. When the viral load samples were collected and tested, the viral load was high.’ (Agnes, 56 years old, female, professional nurse)

‘It was really a challenge managing clients with high viral load, presenting at the health facility when they are almost due to deliver their babies.’ (Bokang, 25 years old, female, professional nurse)

#### Theme 2: Prevention of mother-to-child transmission outcomes

This theme demonstrates that PMTCT service delivery was affected by COVID-19. A loss to follow-up of mother–infant pairs and defaulting and non-adherence to ART emerged as subthemes.

**Subtheme 2.1: Loss to follow-up of mother–infant pairs:** To sever the chain of HIV transmission, treatment continuity is critical for PMTCT clients. Participants found that COVID-19’s restrictive measures meant some clients were lost to follow-up. The participants explained:

‘They were afraid to come to the clinic where most were seeing that they were afraid of contracting COVID-19 and which when you call them to come to the clinic most of them were lost to follow, we did not find them.’ (Lebo, 53 years old, female, 30 years old, female, mentor mother)

‘When we called them to come to the clinic, it was difficult to get them, some had relocated, some of them were lost to follow we did not find them.’ (Promise, 42 years old, female, mentor mother)

**Subtheme 2.3: Defaulting and non-adherence to antiretroviral treatment:** Participants also stated that some PMTCT clients were either defaulting or not adhering to prescribed ART regimens, as the following extracts illustrate:

‘We had an experience of HIV positive PMTCT mothers who were defaulting their ART medicines.’ (Amukelani, 30 years old, female, mentor mother)

‘Some clients would simply not adhere to their prescribed ARV medicines. Some indicated that they were overwhelmed by challenges associated with the COVID-19 pandemic, making it difficult to adhere to their schedules of taking their ARVs.’ (Johanna, 52 years old, female, professional nurse)

### Effects of COVID-19 on prevention of mother-to-child transmission service delivery

This superordinate theme reflects interruptions to PMTCT services were attributed to the pandemic. Two themes emerged, namely PMTCT service disruptions and interruptions to infant HIV testing.

#### Theme 3: Prevention of mother-to-child transmission service disruptions

Participants stated that PMTCT service disruptions occurred, affecting both PMTCT mothers and their infants.

**Subtheme 3.1: Late antenatal care bookings:** According to the participants, some PMTCT clients feared contracting COVID-19, and they were consequently hesitant to visit healthcare facilities. This resulted in late ANC bookings. The following extracts demonstrate these findings:

‘The PMTCT mothers feared contracting COVID-19, hence were not comfortable to visit healthcare facilities and this related in late antenatal care booking.’ (Elisa, 54 years old, female, professional nurse)

‘There was generally a reduction in the number of pregnant mothers coming for antenatal care booking. Those that came, in most cases booked late for antenatal care services-long after 20 weeks gestation.’ (Alina, 38 years old, female, professional nurse)

**Subtheme 3.2: Low client flow at prevention of mother-to-child transmission settings:** Participants reported low client flow at PMTCT facilities during COVID-19, as reflected below:

‘Mm’ during COVID-19 it was a difficult time because PMTCT clients were afraid to bring their children to the clinic, so we had stats that was very low.’ (Abigail, 38 years old, female, mentor mother)

‘There was a reduction in the client flow in both the antenatal and postnatal service points due to fear of contracting COVID-19. In general, there was encouragement that the healthcare facilities should be decongested.’ (Thandi, 51 years old, female, professional nurse)

#### Theme 4: Interruptions to infant HIV testing

Some challenges emerged relating to caring for HIV-exposed infants. Delays in conducting replace with deoxyribonucleic acid-polymerase chain reaction (DNA-PCR) testing in HIV-exposed babies, and HIV-exposed babies’ loss to follow-up emerged as prominent themes.

**Subtheme 4.1: Delays in conducting DNA-PCR testing in HIV-exposed babies:** As a result of COVID-19 lockdown measures, there were delays in PCR testing for HIV-exposed infants, as mentioned below:

‘The clients feared getting ill from COVID-19, even during follow-up phone call, they would express the fear of contracting COVID-19 and unwillingness to bring their babies to the health facility. This resulted in a lot of delays in testing babies for HIV as well as non-adherence to the PMTCT protocols.’ (Sharon, 25 years old, female, professional nurse)

‘They didn’t come to the clinic and other children when doing the PCR later we only found that they tested positive, and others, their mothers were busy breastfeeding them and not taking treatment.’ (Thandi, 51 years old, female, professional nurse)

‘Most of the patients were not coming, most things were not done according to schedule or protocol, since clients did not come to the clinics, they missed their PCR testing and treatment.’ (Mammy, 51 years old, female, mentor mother)

**Subtheme 4.2: HIV-exposed babies’ loss to follow-up:** Participants also indicated that in some instances, HIV-exposed babies could not be traced back to care, as shown in the following extracts:

‘During COVID they didn’t come and after COVID-19 we only found that we have lost many children that way. We were supposed to do their PCR they didn’t come to the clinic.’ (Sharon, 25 years old, female, professional nurse)

‘Because of COVID-19 and related restrictions, it was difficult to track. When tracking activities were initiated, we noted that a lot of babies had not turn up for follow-up visits, it was then that we started following up these clients in the community.’ (Palesa, 48 years old, female, professional nurse)

### Clinical barriers

This theme illustrates that the disrupted clinical care environment affected PMTCT service delivery. The COVID-19 pandemic distracted from the enabling environment necessary to provide quality care. Two themes emerged, namely resource-related barriers and psychological barriers.

#### Theme 5: Resource-related barriers

This theme emphasises the inadequacy of human resources caused by disruptions emanating from the effects of COVID-19. The availability and adequacy of human resources play a critical role in providing an enabling environment necessary for PMTCT service provision. Two subthemes linked to resource-related barriers emerged, namely a shortage of staff and re-assignment of staff.

**Subtheme 5.1: Shortage of staff:** The shortage of staff reported in this study was attributed to absenteeism from COVID-19-related infections, as discussed in the following extracts:

‘There was also a shortage of staff during that time as everyone was getting sick from COVID-19, so it was another thing.’ (Luthando, 50 years old, female, professional nurse)

‘Most of us were coming to work but because of COVID some were staying at home because they were affected by COVID so most of the time we find out that we were short-staffed because of COVID.’ (Agnes, 56 years old, female, professional nurse)

**Subtheme 5.2: Re-assignment of staff:** Participants also indicated that inexperienced nurses had to be assigned to work in the PMTCT setting, for example, in the labour ward, because of staff shortages. This scenario, according to study participants, compromised the quality of care. The following extracts attest that:

‘During COVID-19 It was, it was difficult because of the shortage of staff there yes, we would be two professional nurses on duty and probably one of them was not a midwife, making it difficult to provide services for PMTCT clients.’ (Bokang, 25 years old, female, professional nurse)

‘The situation was challenging, we were just assigned to work in the maternity, even without prior experience because there was a shortage of nurses.’ (Palesa, 48 years old, female, professional nurse)

#### Theme 6: Psychological barriers

The psychosocial barriers participants reported referred to the factors negatively affecting the healthcare environment. These included the fear of contracting COVID-19 and the frustrating and stressful work environment.

**Subtheme 6.1: Fear of contracting COVID-19:** Often, participants found themselves overwhelmed by anxiety and fear. These psychological effects acted as a barrier in service provision, according to the care providers:

‘During COVID-19 it was bad because most of the staff members were sick, this caused a lot of anxiety and fear to us as nurses. Some of us feared being in the hospital environment since we thought we could get infected from patients.’ (Thandi, 51 years old, female, professional nurse)

‘I was afraid and was always anxious thinking that I will contract COVID-19 and spread it to my family. This was emotionally draining, and it was difficult to have a peace of mind. Because of the fear most nurses tried to do task fast, care was then compromised.’ (Elisa, 54 years old, female, professional nurse)

**Subtheme 6.2: Frustrating and stressful work environment:** Participants perceived the work environment to be frustrating and challenging because of the difficulties COVID-19 created:

‘Um the problem was I working in labour ward, the patients we were attending to had not been vaccinated. So, this created additional stress since we were working with the unknown, not knowing whether the patient has COVID-19 or not.’ (Alina, 38 years old, female, professional nurse)

Another participant shared:

‘Mm it was so frustrating, there was increased workload, we also had shortage of staff, some people were sick as they had contracted COVID-19. The work environment was no longer pleasant, instead, it was frustrating.’ (Luthando, 50 years old, female, professional nurse)

## Discussion

The study’s findings indicated that the COVID-19 pandemic’s restrictive measures negatively impacted PMTCT service delivery. Participants reported various service disruptions and PMTCT clients faced challenges in adhering to scheduled clinic visits; this affected PMTCT outcomes.

The findings reflected delays in conducting DNA-PCR testing for HIV-exposed babies or missed HIV testing dates. Therefore, UNICEF^15^ promotes an innovative strategy termed ‘the early infant diagnosis surge strategy’, where there is a line listing and mapping of all HIV-exposed infants who missed their first and second HIV tests. The strategy used healthcare providers to collect infant testing samples and viral load samples from clients who missed clinic visits. In addition, Tomi et al.^23^ advocate for the implementation of home-based dried blood sample collection to increase early infant diagnosis services for HIV-exposed infants; both for adoption during the COVID-19 pandemic and as a sustainable strategy to enhance PMTCT outcomes.

This study’s participants reported that PMTCT clients either defaulted treatment, had treatment interruptions or were lost to follow-up. Non-adherence to treatment has negative implications for the health and wellbeing of HIV-positive mothers, as well as that of their infants or unborn children. These findings are similar to those of a Ugandan study by Kiragga et al.,^24^ where HIV-positive women initiated in HIV care under option B+ and later disengaged from care. In South Africa, PCR testing at birth declined significantly during the first months of COVID-19 and there were some degree of interruptions in PMTCT services during the pandemic.^25^ The non-adherence to scheduled clinic visits or reduced access to PMTCT services established in this study were attributed to transport challenges and the fear associated with contracting COVID-19 as afflicted individuals would expose others visiting the healthcare facilities. Similar findings were observed in other studies by Hogan et al.^26^ and Wanyana et al.,^27^ where the negative impact of the pandemic was found.

According to the WHO,^28^ disruptions in PMTCT service delivery have been reported in 36 countries globally because of COVID-19 lockdown measures. Related findings were established in a study by Flanagan et al.,^14^ which offered empirical evidence of COVID-19’s effect on PMTCT service interruptions and the resultant impact on maternal and paediatric health. UNICEF^15^ further indicates that infant testing is a critical part of the PMTCT care cascade and would be affected by such service disruptions. While this study found COVID-19 impacted mother-infant follow-ups, a PMTCT study in Ethiopia, conducted away from the COVID-19 environment, documented a lower rate of loss to follow-up.^29^

This study reported that there was generally low client flow in PMTCT settings during the pandemic. Similarly, evidence from Uganda^30^ and Ethiopia^31^ reflected reduced antenatal attendance during the first months of the pandemic. Another study conducted in Zimbabwe reported low PMTCT coverage and low attendance at both antenatal and postnatal clinics, attributed to individuals’ fear of contracting the virus at health facilities and transport challenges.^8^ In contrast to this study’s findings, Siedner et al.^32^ found no decrease in adults’ clinic visits at the start of the level five COVID-19 lockdown or related HIV care. However, the same study established a significant drop in child healthcare visits. This study found that HIV-exposed babies missed DNA-PCR testing as they could not adhere to scheduled visits. The findings are supported by evidence from China,^33^ where there was a decrease in paediatric healthcare visits. This study’s findings are thus not unique; as the evidence demonstrates, during previous disease epidemics in sub-Saharan Africa, reductions in access to primary healthcare services were similarly reported, including reduced facility-based deliveries and child healthcare access.^34^

The study established that COVID-19 psychologically affected the participants involved in PMTCT service provision. Evidence demonstrated that in order to enhance the quality of healthcare service provision, the ministries of health would need to invest in providing mental health support for its workforce.^35^ Turale and Nantsupawat^36^ concurred that nursing during COVID-19 had a significant impact on the mental health of nurses and called for the ministries of health and governments to seriously consider investing in nursing. These findings are similar to those of a study in Italy by Chirico and Magnavita^37^ that established healthcare workers’ psychological and mental health was affected by the pandemic, resulting in anxiety, depression and sleep disturbances. Shaukat et al.^38^ indicate that higher levels of stress, fear and anxiety are experienced by nurses because they spend more hours with patients compared to other healthcare professionals. To address this challenge, reduce the psychological effects on professional nurses and mentor mothers’ experience and facilitate the continuity of healthcare services during pandemics, Moyo et al.^8^ advocate for a robust support system for service providers. Furthermore, Tomlin et al.^39^ emphasise the importance of mental health and wellbeing in the context of the challenging COVID-19 environment.

The shortage of human resources in the study’s setting implies it was difficult to offer routine PMTCT service delivery. The shortage of staff in healthcare settings is not unique to this study; similar findings were noticed by McNeill^40^ in the United States. A related study in Zimbabwe by Moyo et al.^8^ found an increased workload during the pandemic, and the work environment was perceived to be busy and challenging. In addition, Turale and Nantsupawat^35^ determined that nurses had to work extra shifts during the pandemic because of acute nursing shortages. To enhance service provision, O’Donovan and McAulife^41^ call for an effective structured support system for nurses involved in service provision during pandemics and assigned to new and challenging roles.

### Limitations

The study was limited to three primary healthcare facilities of Tshwane and not the whole of Tshwane district and therefore findings could not be generalised. Despite the highlighted limitations, this study provides evidence of the interruptions of the PMTCT service delivery during the COVID-19 outbreak.

## Conclusion

The study’s findings demonstrated that the restrictive measures meant to curb the spread of COVID-19 impacted PMTCT service delivery and PMTCT outcomes. The findings emphasise the importance of epidemic or pandemic preparedness and responsiveness, considering strategies to enhance service continuity and prevent disruptions. The development and implementation of innovative strategies are critical to enhance PMTCT service delivery during health emergencies.
